# Brain cortical characteristics of lifetime cognitive ageing

**DOI:** 10.1007/s00429-017-1505-0

**Published:** 2017-09-06

**Authors:** Simon R. Cox, Mark E. Bastin, Stuart J. Ritchie, David Alexander Dickie, Dave C. Liewald, Susana Muñoz Maniega, Paul Redmond, Natalie A. Royle, Alison Pattie, Maria Valdés Hernández, Janie Corley, Benjamin S. Aribisala, Andrew M. McIntosh, Joanna M. Wardlaw, Ian J. Deary

**Affiliations:** 1 Centre for Cognitive Ageing and Cognitive Epidemiology, University of Edinburgh, 7 George Square, Edinburgh, EH8 9JZ UK; 2 Department of Psychology, University of Edinburgh, Edinburgh, UK; 3 Scottish Imaging Network, A Platform for Scientific Excellence (SINAPSE) Collaboration, Edinburgh, UK; 4 Brain Research Imaging Centre, Neuroimaging Sciences, University of Edinburgh, Edinburgh, UK; 5 Department of Computer Science, Lagos State University, Lagos, Nigeria; 6 Division of Psychiatry, University of Edinburgh, Edinburgh, UK

**Keywords:** Ageing, Intelligence, MRI, Cortex, Thickness, Surface area

## Abstract

Regional cortical brain volume is the product of surface area and thickness. These measures exhibit partially distinct trajectories of change across the brain’s cortex in older age, but it is unclear which cortical characteristics at which loci are sensitive to cognitive ageing differences. We examine associations between change in intelligence from age 11 to 73 years and regional cortical volume, surface area, and thickness measured at age 73 years in 568 community-dwelling older adults, all born in 1936. A relative positive change in intelligence from 11 to 73 was associated with larger volume and surface area in selective frontal, temporal, parietal, and occipital regions (*r* < 0.180, FDR-corrected *q* < 0.05). There were no significant associations between cognitive ageing and a thinner cortex for any region. Interestingly, thickness and surface area were phenotypically independent across bilateral lateral temporal loci, whose surface area was significantly related to change in intelligence. These findings suggest that associations between regional cortical volume and cognitive ageing differences are predominantly driven by surface area rather than thickness among healthy older adults. Regional brain surface area has been relatively underexplored, and is a potentially informative biomarker for identifying determinants of cognitive ageing differences.

**Electronic supplementary material:**

The online version of this article (doi:10.1007/s00429-017-1505-0) contains supplementary material, which is available to authorized users.

## Introduction

On average, several important cognitive functions exhibit a mean decline with age in adulthood (e.g., Singh-Manoux et al. 2012; Hedden and Gabrieli 2004). Scores on various tests of specific cognitive functions are generally well correlated and a single measure of general cognitive ability or general fluid intelligence tends to explain the much of this variation in individual test scores (Jensen 1998; Johnson et al. 2008). A large component of age’s effect is on general cognitive ability (Salthouse 2004; Tucker-Drob et al. 2014), and its age-related declines limit everyday functioning, independence, and quality of life (Tucker-Drob, 2011; Plassman et al. 2008; Bárrios et al. 2013). Although there is mean age-related change, between-person differences in cognitive functioning from youth to older age are relatively stable; the correlation between youth and older age intelligence is typically ~0.60 to 0.70 (reviewed in Deary 2014). Importantly though, the correlation is not perfect. Therefore, discovering factors that might explain variance in relative cognitive change from youth to older age would help our understanding the determinants and mechanisms of cognitive ageing. Here, we investigate which brain cortical measures, at which loci are pertinent for lifetime ageing differences in general cognitive ability.

The cortex of the human brain changes in volume, surface area, and thickness throughout the life course (e.g., Storsve et al. 2014; Lemaitre et al. 2012; Hogstrom et al. 2013; Schnack et al. 2015; Ziegler et al. 2012). These alterations are thought to explain—at least in part—age-related changes in the cognitive abilities that the brain, as a whole, underpins (Raz and Rodrigue 2006; Salthouse 2011). Older age is consistently associated with greater reductions in the brain’s dorsal frontal, parietal, and lateral temporal volume, thickness, and surface area, although evidence regarding motor, somatosensory, and visual cortices is inconsistent (Dickie et al. 2015; Fjell et al. 2014; Hogstrom et al. 2013; reviewed in Raz and Rodrigue 2006 and in McGinnis et al. 2011). There is some evidence for age-related cortical thickening around the medial orbitofrontal/anterior cingulate area (Salat et al. 2004; Fjell et al. 2009, 2014). Others report that individual cortical regions exhibit age-related changes in surface area that are no larger than those measured across the whole brain (Lemaitre et al. 2012), implying a lack of anatomical specificity and an ageing effect that globally affects the cortex. Studies comparing associations among all three cortical measures are scarce, and it is also unclear whether regional volumetric changes in the brain’s cortex are driven mainly by thickness (Storsve et al. 2014), or by surface area (Im et al. 2008; Dickerson et al. 2009; Dotson et al. 2015). Importantly, well-powered data on the way that covariances among cortical characteristics are patterned across the cortical mantle are lacking in older participants.

Cortical thickness and cortical surface area are both heritable, but genetically independent (Chen et al. 2015; Eyler et al. 2012; Panizzon et al. 2009; Winkler et al. 2010). They exhibit distinct change trajectories across the life course (Raznahan et al. 2011; Storsve et al. 2014). Therefore, it is plausible that they might have differential importance for outcomes such as cognitive ageing. Fleischman et al. (2014) examined cross-sectional associations of cognitive functioning with brain cortical volume, thickness, and surface area in older age (*n* = 186, range 65–100 years). They reported no cortical correlates of global cognitive function across regional volume, surface area, or thickness.

To study cognitive and brain ageing, one needs to have measures that assess their changes across time. With respect to the brain, longitudinal data are highly valuable, but where prior brain data from earlier in life are unavailable, cross-sectional neuroimaging studies tend to correct for head size in the form of intracranial volume (ICV), which also provides an estimate of maximal healthy brain size (e.g., Royle et al. 2013, their Fig. 2). However, such studies are rarely able to make the corresponding correction in the cognitive domain; that is, they tend not to have any assessment of prior cognitive ability from which the older age cognitive test scores can be compared to measure cognitive ageing. Even fewer are able to analyse changes in cognitive function across many decades (Deary 2014). Thus, most studies are unable to identify the portion of variance in older-age cognitive ability that can be ascribed to pre-existing differences in the study sample. In the current study, 604 participants provided brain MRI data at age 73 years, and measures of cognitive ability, both in youth and ~60 years later. Well-powered studies are important in this area, especially since neuroimaging samples tend to include relatively few individuals over 70 years of age (Fleischman et al. 2014). We examined the regional cortical characteristics of lifetime cognitive ageing differences, from 11 to 73 years. We investigated which aspects, in which brain cortical loci, are linked to the relative maintenance or improvement in cognitive function from childhood to older age. Based on information on the proposed cortical loci of intelligence differences (Jung and Haier, 2007; Colom et al. 2010; Deary et al. 2010; Barbey et al. 2012), we hypothesised that greater lateral frontal, parietal, and temporal regions—and not those related to motor and somatosensory processes—would be most strongly associated with less relative lifetime cognitive change. Using a vertex-wise method across the cortical surface in the same sample, we previously reported no associations between cortical thickness and intelligence in older age, after accounting for intelligence in youth (Karama et al. 2014). In the present study, we go further using a region-of-interest approach to measure and compare associations between cortical volume, surface area, and thickness and relative lifetime change in cognitive ability.

## Materials and methods

### Participants

In June 1947, almost all age 11 school children in Scotland sat in the Moray House Test No. 12 (MHT) as a nationwide survey of intelligence. Between 2004 and 2007, 1091 (543 female) community-dwelling older adults, all born in 1936, were recruited to form the Lothian Birth Cohort 1936 (LBC1936) study for Wave 1 testing of cognitive and health testing (Deary et al. 2007). Wave 2 testing took place at age ~73, and combined the previously administered cognitive and physical battery with a comprehensive structural MRI examination (Deary et al. 2012; Wardlaw et al. 2011). From the 866 participants who attended Wave 2, 604 participants provided the following complete data: MRI brain scan, contemporaneous cognitive data, an MHT score from age 11, and a score of 24 or above on the Mini-Mental State Examination (MMSE; Folstein et al. 1975), and reported no diagnosis of dementia. The Multi-Centre Research Ethics Committee for Scotland (MREC/01/0/56) and Lothian Research Ethics Committee (LREC/2003/2/29) approved use of the human subjects in this study; all participants provided written informed consent and these have been kept on file.

### Cognitive testing

The MHT was concurrently validated at age 11 against the Stanford Revision of the Binet intelligence test, with which it correlated about 0.8 (Deary et al. 2000). A measure of general fluid intelligence at LBC1936 Wave 2 (age ~73 years) was derived from the first unrotated component of a principal components analysis of scores on Matrix Reasoning, Block Design, Digit Span Backward, and Letter-Number Sequencing subtests from the Wechsler Adult Intelligence Scale, 3^rd^ UK Edition (Wechsler, 1998). This first component explained 55.3% of the variance across the tests, and all loadings were >0.73. Intelligence exhibits notable stability across the life course, and associations between youth and older age cognitive ability measured in this way typically range from 0.60 to 0.70 (reviewed in Deary 2014).

### MRI acquisition

Full details of the whole-brain MRI structural protocol are available in an open-access article (Wardlaw et al. 2011). Briefly, participants were scanned on a GE Signa Horizon 1.5 Tesla HDxt clinical scanner (General Electric, Milwaukee, WI, USA). Acquisition comprised T2-, T2*-, and FLAIR-weighted axial scans, and a high-resolution 3D T1-weighted volume sequence acquired in the coronal plane (voxel dimensions 1 × 1 × 1.3 mm).

### MRI analysis

ICV was measured using a validated semi-automated multispectral fusion method, guided by intensities from a series of combined sequences (T1-, T2-, T2*-, and FLAIR-weighted) for segmentation (Valdés Hernández et al. 2010). All segmented images were visually examined for accuracy on anonymised scans to correct errors (Wang et al. 2012). Cortical reconstruction and volumetric segmentation of T1-weighted images were performed with FreeSurfer v5.3 (http://surfer.nmr.mgh.harvad.edu/) using the Desikan-Killiany atlas (Desikan et al. 2006). The processing steps involved removal of non-brain tissue using a hybrid watershed/surface deformation procedure (Segonne et al. 2004), intensity normalisation (Sled et al. 1998), tessellation of the grey-white matter boundary and automated topology correction (Fischl et al. 2001; Segonne et al. 2007), and surface deformation following intensity gradients to optimally place the grey–white and grey–CSF borders at the location where the greatest shift in intensity defines the transition to other tissue classes (Fischl and Dale 2000). The cortical surfaces were then inflated and registered to a spherical atlas that used individual cortical folding patterns to match cortical geometry across subjects (Fischl et al. 1999a, b; Desikan et al. 2006; Fischl et al. 2004). This method used both intensity and continuity information from the entire MRI volume to produce representations of cortical thickness and calculated as the closest distance from the grey–white matter boundary to the grey–CSF boundary at each vertex on the tessellated surface (Fischl and Dale 2000).

**Fig. 1:**
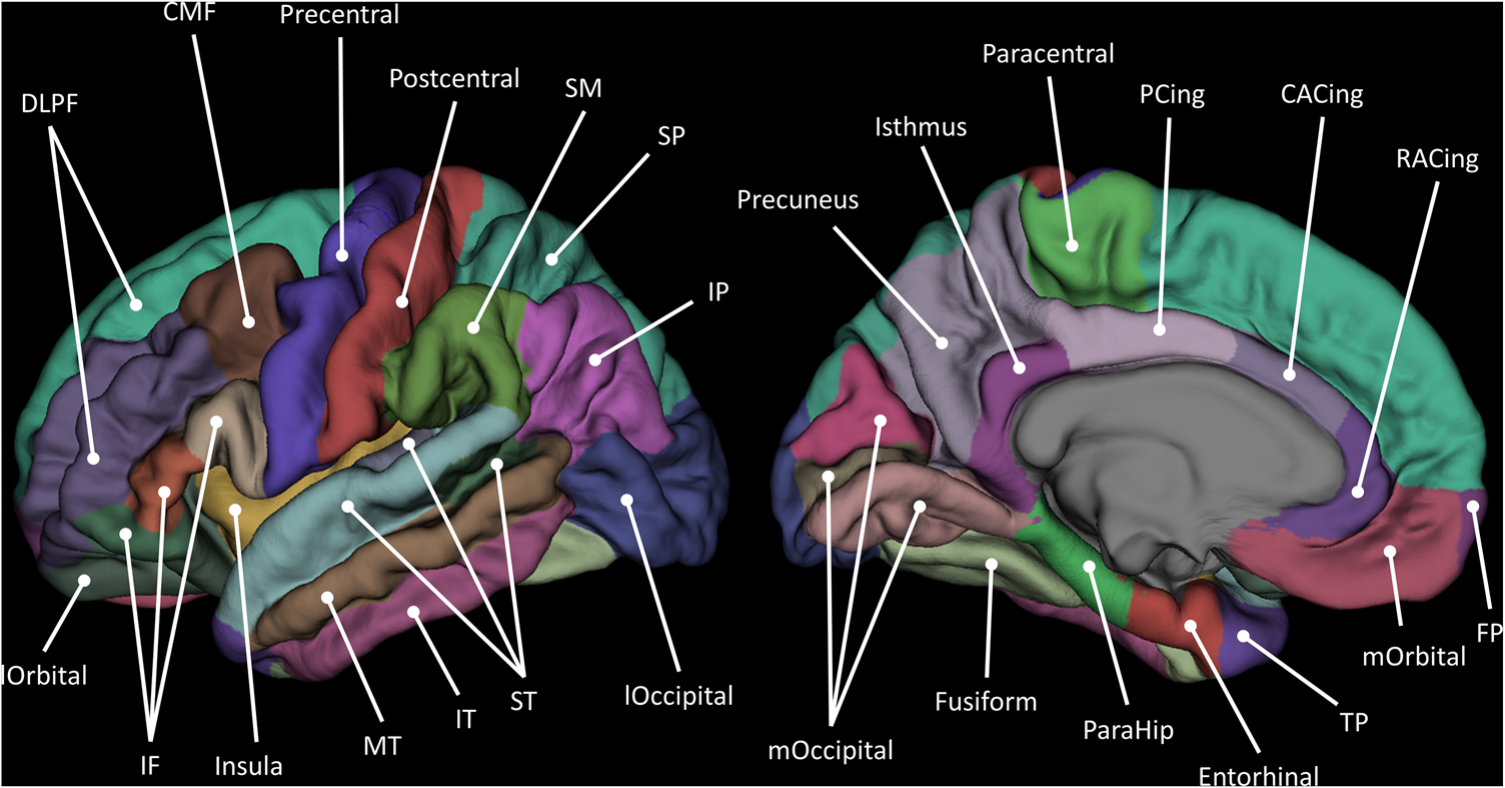
Schematic of parcellated cortical regions according to Desikan et al. (2006). *FP* frontal pole, *DLPF* dorsolateral prefrontal, *IF* inferior frontal, *lOrbital* lateral orbitofrontal, *mOrbital* medial orbitofrontal, *CMF* caudal middle frontal, *RACing* rostral anterior cingulate, *CACing* caudal anterior cingulate, *TP* temporal pole, *ST* superior temporal, *MT* middle temporal, *IT* inferior temporal, *ParaHip* parahippocampal, *SP* superior parietal, *IP* inferior parietal, *PCing* posterior cingulate, *lOccipital* lateral occipital, *mOccipital* medial occipital. See Supplementary Material for further information

Regional measures of cortical thickness, therefore, represented the mean distance from the grey–white matter boundary to the grey–CSF boundary within a defined cortical region. Surface area measures at the grey–white matter boundary were derived from the sum of all triangular tessellations in each anatomical parcel, with volume as the product of surface area and thickness. All segmentations underwent visual quality control which identified and removed (listwise) instances of general segmentation failure or deficiencies in tissue identification (including those with cortical lesions) often caused by movement, and removed individual parcels (casewise) affected by minor skull strip issues and regional boundary positioning errors. A maximum *n* of 568 participants provided cortical volume, surface area, and thickness for 27 regions per hemisphere (Fig. 1), alongside complete cognitive data. Further details on the parcellation schema and selection of regions for analysis are provided in Supplementary Methods, Table S1, and in Cox et al. (2014). In a supplementary analysis (to illustrate associations which are agnostic to sulcal/gyral boundaries of the Desikan–Killiany parcellation), we registered the vertices of each participant to the FreeSurfer average pial surface (using the qcache command in FreeSurfer), applying a smoothing kernel of 20 mm full width at half maximum.

### Statistical analysis

The MHT score from age 11 (out of a maximum 76) was converted to an intelligence quotient scale (mean = 100, SD = 15) and corrected for age in days at time of testing. Cognitive ageing scores were then computed as the residuals of a linear regression between the intelligence measures from age 11 (MHT) and 73 (the general component from the Wechsler subtests), corrected for sex and age in days at testing in older age. This score (a standardised residual with a mean of 0 and SD of 1) represented the degree to which individuals were performing better or worse at age 73, relative to their ability at age 11, in the context of other members of the cohort. First, descriptive associations among uncorrected whole-brain and regional cortical measures were examined—we analysed the degree to which global and local measures of volume, cortical surface area, and thickness were correlated. Next, regional MRI measures—expressed as a proportion of ICV and corrected for sex and age in days at time of MRI—were used to calculate Pearson’s product-moment correlation coefficients between the three brain cortical characteristics (volume, surface area and thickness) and cognitive ageing differences per ROI in each hemisphere. In the supplementary analysis, we used the SurfStat toolbox (http://www.math.mcgill.ca/keith/surfstat) for Matrix Laboratory R2014a (The MathWorks Inc., Natick, MA) to conduct regressions at each vertex for volume, thickness, and surface area on cognitive ageing (already corrected for age and sex), correcting for ICV. Results were corrected for multiple comparisons using False Discovery Rate (FDR; Benjamini and Hochberg 1995) applied for volume, surface area, and thickness, separately (given the large degree of collinearity and consequent redundant hypothesis testing). All analyses were conducted in R (version 3.03; http://www.r-project.org) and cortical surface ROI visualisations used the Liewald–Cox Heatmapper tool (http://www.ccace.ed.ac.uk).

## Results

**Fig. 2:**
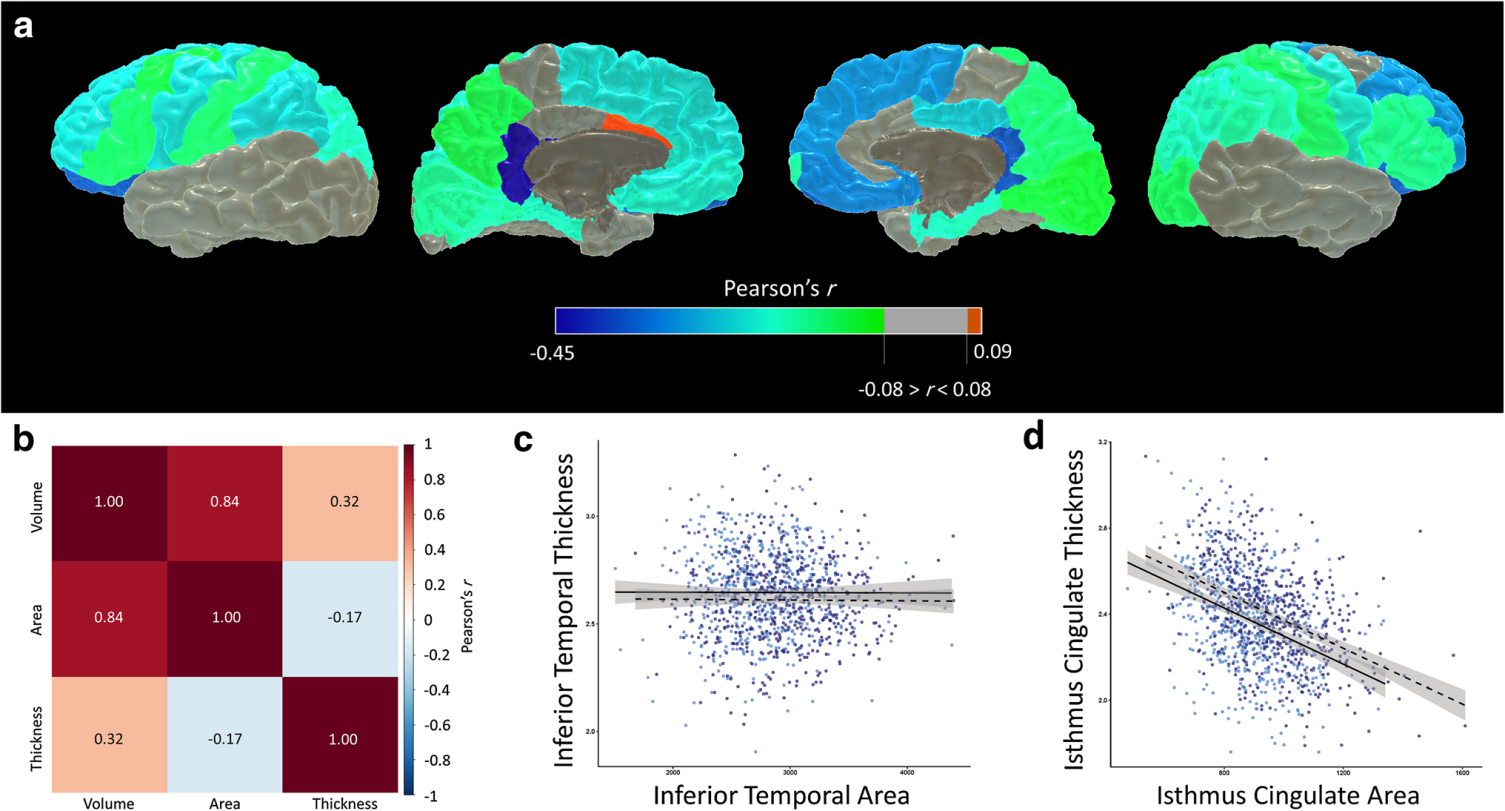
**a** Regional associations between raw cortical surface area and thickness; **b** correlation matrix of associations between global volume, surface area, and thickness; **c** absence of association between cortical surface area and thickness in inferior temporal gyrus; **d** strongest negative association between cortical surface area and thickness is exhibited in the isthmus cingulate gyrus. For panels **c** and **d**, *light blue points* and *solid regression lines* (with *shaded* 95% confidence intervals) denote *right sided* measures, and *dark blue points* and *broken regression lines* indicate *left sided* measures

Table 1 shows general descriptive statistics for the LBC1936 participants. For illustrative purposes, and to aid comparison with samples from other studies, we also report other characteristics reported during medical interview at Wave 2: 10.32% reported a diagnosis of diabetes, 49.40% reported receiving a diagnosis of hypertension, 42.21% had a diagnosis of hypercholesterolemia, and 27.25% had a history of cardiovascular disease. Consistent with prior reports, intelligence at age 11 and age 73 years was significantly correlated (*r* = 0.553, *p* < 0.001). Total brain volume was significantly associated with total cortical surface area (*r* = 0.837, *p* < 0.001) and with average cortical thickness (*r* = 0.315, *p* < 0.001). Total surface area and average thickness were significantly negatively correlated (*r* = −0.175, *p* < 0.001). Further examination of the relationship between surface area and thickness revealed significant heterogeneity across the cortical mantle (Fig. 2 and Table S2). Surface area and thickness were most strongly related (negatively) in bilateral isthmus cingulate, and dorsolateral and orbitofrontal areas (*r* = −0.223 to −0.454, *p* > 0.001), and were associated positively in the caudal anterior cingulate (*r* = 0.087, *p* = 0.037), though the latter did not survive FDR correction. Thickness and surface area were not significantly associated in the left and right lateral temporal areas (pole, superior, middle, and inferior temporal gyri), fusiform, paracentral, or right anterior cingulate regions (all *r* values <0.078, all *p* values >0.063).

**Table 1:** Lothian Birth Cohort 1936 characteristics

Age at MRI, *M* (SD) years	72.64 (0.72)
Female, *n* (%)	284 (47.02%)
Matrix Reasoning, *M* (SD)	13.51 (4.9)
Block Design, *M* (SD)	34.34 (10.01)
Digits Backwards, *M* (SD)	7.85 (2.27)
Letter-number Sequencing, *M* (SD)	10.95 (2.98)
Age 11 IQ	100.802 (15.46)
MMSE, *M* (SD)	28.85 (1.26)
Total brain volume *M* (SD) mm^3^	992114.80 (90778.76)
Cortical volume, *M* (SD) mm^3^	404882.30 (36602.32)
Cortical surface Area, *M* (SD) mm^2^	152948.80 (13925.07)
Cortical thickness, *M* (SD) mm	2.39 (0.11)

Thickness, surface area, and volume measures are computed across all ROIs

*MMSE* Mini-Mental State Examination

**Fig. 3:**
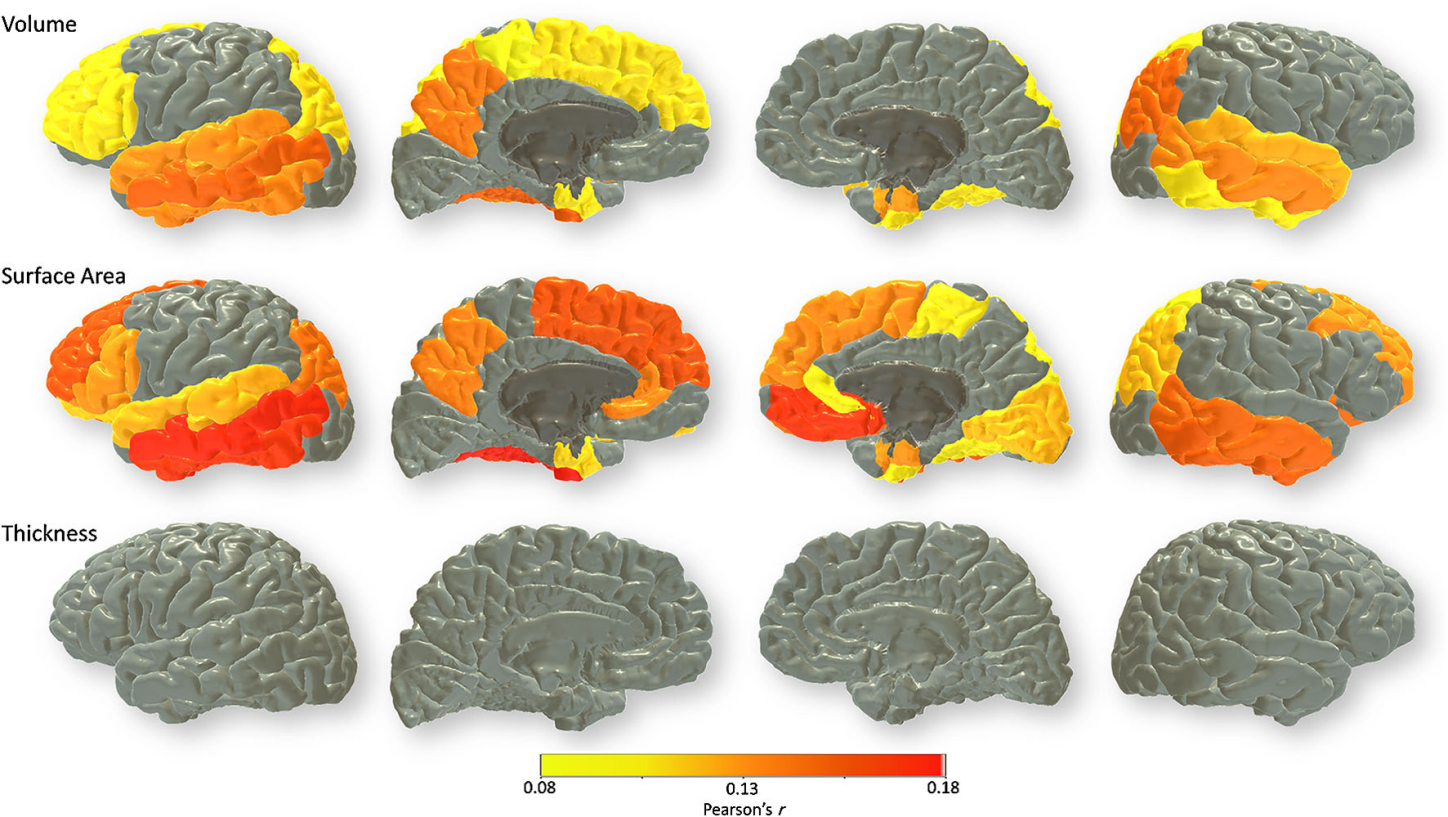
FDR-corrected associations between lifetime cognitive change between 11 and 73 (corrected for age at testing, sex) and brain cortical volume (*top row*), surface area (*middle*), and thickness (*bottom*). Magnitude (Pearson’s *r*) of association is reported for each regional cortical measure (corrected for age at scan, sex, and intracranial volume)

Regional associations between cortical measures and lifetime cognitive ageing differences are shown in Fig. 3 and Table S3. The pattern of associations between cortical regions and cognitive ageing scores was markedly similar for volume and surface area. In contrast, there were only two nominally significant associations across all regions for cortical thickness (right caudal anterior cingulate and right caudal middle frontal; both *r* = −0.087, *p* = 0.039), neither of which survived FDR correction. We found significant associations for brain cortical volume and surface area in the left lateral frontal lobe (dorsolateral *r* = 0.108 and 0.157; and inferior *r* = 0.107 to 0.129), the temporal lobe bilaterally (middle *r* = 0.134 to 0.175; inferior *r* = 0.105 to 0.169; entorhinal *r* = 0.106 to 0.129; and fusiform *r* = 0.099 to 0.180), and the inferior parietal lobe (*r* = 0.110 to 0.148), all of which survived correction for multiple testing. There were also FDR-corrected significant associations between lifetime cognitive ageing and surface area in the right dorsolateral (*r* = 0.133), right paracentral lobule (*r* = 0.103), right medial occipital (*r* = 0.094), and bilateral orbitofrontal (medial *r* = 0.090 and *r* = 0.170; and lateral *r* = 0.113 and *r* = 0.146 for left and right, respectively) regions. Supplementary vertex-wise analyses (Figure S1) corroborated the pattern of associations across cortical ROIs, and also that volumetric associations with lifetime cognitive ageing appeared to be driven by surface area rather than thickness. There were, however, some slight differences in that i) some dorsolateral associations for volume and area (particularly the former) did not survive correction for multiple testing in the vertex-wise analysis which were significant at the level of ROIs, and ii) there were small significant clusters for cortical thickness in the anterior temporal lobes in contrast to the non-significant ROI associations.

## Discussion

We found that regional cortical volume and surface area, but not cortical thickness, were associated with cognitive ageing over ~60 years in a large cohort of community-dwelling older adults. By removing variance in later-life intelligence that was attributable to pre-existing differences in intelligence from youth, we identify brain measures that, in older age, are related to the lifetime change intelligence differences, and may thus be valuable for our mechanistic understanding of determinants of cognitive ageing. That is: which cortical features are sensitive to better or worse cognitive performance in older age than one would expect from cognitive performance in youth. Surface area in these cortical regions thus appears to be related to an index of cognitive function which is reliably affected by increasing age (Salthouse 2004; Tucker-Drob et al. 2014), and is an important marker of independence, everyday functioning, and quality of life in older age (Tucker-Drob 2011; Plassman et al. 2008; Bárrios et al. 2013).

Of particular note is the significant, but region-specific pattern of effects for surface area, in contrast to the comparative absence of association in any region for cortical thickness. This suggests that the former drives observed associations between brain volume and cognitive ageing. While the participants may well have experienced a degree of cortical thinning with age (e.g., Jiang et al. 2014, but see Dotson et al. 2015), the non-significant associations with cognitive ageing differences, across all regions, correspond well with entirely null findings in the same cohort using an alternative voxel-wise approach (Karama et al. 2014). We are unaware of any extant studies that analyse relationships between cortical surface area and lifetime cognitive change in the same individuals. Our data indicate that frontal, temporal, and parietal surface area, as opposed to thickness, appears to be a potentially informative cortical biomarker with respect to cognitive ageing differences across the life course, at least in healthy, non-demented older people.

The plausibility of our findings is corroborated by the pattern of observed associations with intelligence. Associations between frontal, parietal, and temporal regions are consistent with the distributed network of cortical regions hypothesised to facilitate intelligence, such as the Parieto-Frontal Integration Theory (P-FIT; Jung and Haier 2007; Colom et al. 2010; Deary et al. 2010; Barbey et al. 2012). Notably, studies reporting trajectories of cortical change in older age frequently include other regions (such as motor and somatosensory areas, e.g., Fjell et al. 2014; Hogstrom et al. 2013; Raz and Rodrigue 2006; McGinnis et al. 2011) that the current data do not identify as being relevant for cognitive ageing differences. This supports an interpretation of these regions as differentially sensitive to lifetime cognitive change—but this does not, of course, preclude the possibility that these regions experience age-related changes which are pertinent to other types of function not measured here.

We also reported the associations between thickness and surface area across the cortical mantle. Well-powered neuroanatomical information on participants in the eighth decade of life is currently sparse. The pattern of associations which we found is highly consistent with those reported by Hogstrom et al. (2013), who examined these same associations across adults of a broader age range (*n* = 322, aged 20–85 years), with fewer older participants (*n* = 116, aged 60–85 years). They also found weak or null associations in the paracentral area, and in superior temporal and parahippocampal regions, and reported the same positive association between cortical thickness and surface area in the left anterior cingulate as was found in the current study. However, in contrast to our findings, their whole-group analysis identified strong associations in middle and inferior temporal areas which were notably absent in our (older aged) group. It is possible that this relationship—which may be relatively stable in earlier life (2/3rds of the Hogstrom sample was aged <60 years)—becomes less stable into the 8th decade, though longitudinal data are required to adequately examine the coevolution of these measures with advancing age among older age groups.

The relative phenotypic independence of cortical thickness and surface area reported herein, and the stronger associations for cortical surface area (versus thickness) with differences in cognitive ageing, are consistent with other data. Both brain measures are heritable but nonetheless genetically independent (Chen et al. 2015; Panizzon et al. 2009; Winkler et al. 2010), and the genetic correlation between neocortical volume and intelligence may be driven by surface area, not thickness (Vuoksimaa et al. 2015). Other age-related markers of brain maturation and ageing, such as hyperintensity volume and microstructural properties, which are important for cognitive ageing (Ritchie et al. 2015a, b) do not seem to co-occur with changes in cortical thickness (Tamnes et al. 2010; Dickie et al. 2016), though more data regarding their coevolution with cortical surface area and volume are required. Furthermore, a post-mortem study across 94 brains (aged 18-93 years) indicated that cortical volume and surface area (but not thickness) were related to neocortical neuron counts (Pakkenberg and Gunderson 1997). The precise neurobiological underpinnings of thickness versus surface area correlations are currently moot, and several avenues have been suggested: cortical surface area (and its greater sensitivity to lifetime cognitive ageing) may be more directly driven than thickness by differences in the proliferation of precursor cells during development; the horizontal >vertical growth and myelination of thalamo-cortical and cortico-cortical tracts (which appear most sensitive to increasing age; Cox et al. 2016a), and the development and ageing of pyramidal cell neuropil (Winkler et al. 2010). It is conceivable that many of these characteristics are affected by multiple aspects of both developmental and ageing processes. Further delineating potential neurobiological mechanisms alongside their timing and determinants constitutes an important challenge for future research into cognitive ageing.

The stronger association between cognitive function and cortical surface area (relative to thickness) was also found in a sample of children (Walhovd et al. 2016). Moreover, when the authors extracted the surface area across all regions which were significantly associated with cognitive ability in children—regions which bear a striking resemblance to those reported herein—the differences in surface area between high and low cognitive ability groups across 974 individuals aged 4–88 were age invariant (i.e., the ‘advantage’ of greater surface area shown by those with higher ability was relatively constant, neither widening nor narrowing with increasing age; Walhovd et al. 2016). Taken together, the phylogenetic principle of maximising surface area, rather than thickness, for the benefit of cognitive capacity (Hogstrom et al. 2013) may apply to selective cortical regions in relation to life course changes in intellectual function, and such differences found in older age may be partly influenced by several important early life factors (Cox et al. 2016b; Walhovd et al. 2016).

One possible explanation for our null findings relating to cortical thickness may be due to our study sample composition; reports have indicated that marked changes in regional brain volume are driven by cortical thickness in pathological states, but not in ostensibly healthy controls. For example, lower cortical thickness (but not surface area) is associated with higher genetic or environmental liability for schizophrenia (Hedman et al. 2016) and with multiple sclerosis (Nygaard et al. 2015). Alterations in medial temporal lobe volumes in Alzhiemer’s disease may be driven by thickness (compared to surface area in healthy controls; Dickerson et al. 2009, though this may not uniquely indicate neurodegenerative illness, Fjell et al. 2014): associations between cortical thickness and surface area were notably null in these regions in our study. No participant in our sample reported a diagnosis of dementia and we additionally employed cognitive screening (MMSE) as a further check. It is possible that some participants were in a prodromal stage of dementia, but our results may not generalise to more representative samples of older adults—particularly in the eighth decade of life during which dementia risk is markedly increased (Matthews and Brayne 2005).

Our study has other limitations. Participants were all recruited from a single area in Scotland (Edinburgh and the Lothians) and had a narrow age range. Though it may be argued that this removes the important potential confounds of age (Hofer and Sliwinski 2001), cultural heterogeneity, and genetic variability, this also restricts the generalisability of these findings to other populations. Our findings are correlational in nature and, therefore, cannot be taken as strong evidence that cortical surface area in all significant regions contributes to lifetime change in intelligence differences. The same effect could also come about if a single mechanism jointly affects all regions identified, but only some regions are directly involved in intellectual performance. In addition, though it is highly beneficial to have a measure of intelligence from youth and older age, we are unable to comment on cause and effect: when or how cognitive changes occur across the life course, and their association with early life brain structure beyond maximal healthy brain size (ICV) is unclear.

These findings highlight the differential relevance of cortical characteristics for lifetime ageing differences in intellectual ability. The rarity of intelligence scores spanning ~60 years and the large sample of well-characterised participants in a previously under-represented age group allow a valuable examination of the possible biological bases of cognitive ageing. We cautiously posit that cortical surface area—which has received comparatively a little attention with respect to cognitive ageing—could be a valuable biomarker for understanding the determinants of age-related brain and cognitive changes. Mapping the coevolution of various cortical measures and cognitive trajectories into older age are important topics for future research into pathological and non-pathological ageing.

## Electronic supplementary material

Below is the link to the electronic supplementary material.

Supplementary material 1 (DOCX 1565 kb)
