# Supplementary material for: Brain cortical characteristics of lifetime cognitive ageing
Source: Brain Struct Funct. 2017 Sep 6;223(1):509–18. doi: 10.1007/s00429-017-1505-0 (PMC5772145; doi:10.1007/s00429-017-1505-0)
Supplement: Supplementary file 1 — Supplementary material 1 (DOCX 1565 kb) [file 429_2017_1505_MOESM1_ESM.docx]

**Supplementary Material**

**Supplementary Methods:**

Eleven individual regions of interest derived according to the default settings and parcellation schema in Freesurfer (Desikan *et al.,* 2006) were combined (summed for volume and surface area, averaged for thickness) into four regions per hemisphere as follows: inferior frontal gyrus (pars opercularis, pars orbitalis, pars triangularis), dorsolateral prefrontal cortex (rostral middle frontal, superior frontal), superior temporal gyrus (banks of the superior temporal sulcus, transverse temporal, superior temporal) and medial occipital (pericalcarine, cuneus, lingual), giving a total of 27 regions for analysis per hemisphere (Figure 1). The theoretical justification for doing so is outlined below:

*Table S1*. Schema and notes on combining Desikan atlas regions.

| **Lobe** | **Native Desikan Regions** | **Macro-Region** |
| --- | --- | --- |
| Frontal | Superior Frontal + Rostral Middle Frontal | Dorsolateral Prefrontal Cortex^a^ |
|  |  |  |
|  | Pars Opercularis + Triangularis + Orbitalis | Inferior Frontal Gyrus^b^ |
|  |  |  |
| Temporal | Superior Temporal + Transverse Temporal + Banks Superior Temporal | Superior Temporal Gyrus^c^ |
|  |  |  |
| Occipital | Pericalcerine + Lingual + Cuneus | Medial Occipital^d^ |
|  |  |  |

^a^Rajkowsa & Goldman-Rakic (1995; figure 12); also discussed in Cox *et al.* (2014). The cellular architecture of BA9/46 is common to both superior and middle frontal gyri. Technically, one might argue that the posterior portion of both middle and superior frontal gyri should be excluded from this measure. While the middle frontal gyrus is divided in this way by the Desikan atlas, the parcellation of the superior frontal region is not (see Figure 1), constituting a minor limitation of the current method.

^b^ Though there is literature supporting distinct functional contributions of the *pars* of the inferior frontal gyrus, these regions are small, difficult to accurately identify and show a weak gryal:cytoarchitectural correspondence (see Cox *et al*. 2014 for a detailed discussion). Moreover, for associations with intelligence, we judge that the possible functional difference of the individual *pars* do not lead us to hypothesise that any one would have stronger association than others.

^c^ As per the inferior frontal gyrus, the Banks STS and Transverse Temporal Gyrus are small and difficult to accurately identify – the latter not being directly bound by specific sulci. Given the lack of clear hypotheses regarding associations between these small regions and cognitive ageing, they were combined into the superior temporal gyrus.

^d^ The cuneus and pericalcerine cortex are both involved in relatively basic visual processing, with the lingual gyrus most consistently related to the visual processing of letters, and so might be combined if the hypothesis of interest is not directly related to different aspects of these functions. For example, one might not hypothesise that different branches of the tracts detailed in Catani *et al.* (2003) are differentially linked to general cognitive ability. Forkel *et al*. (2014) indicates that grosser regions might be equally appropriate for identifying regions with shared occipito-frontal connections.

**References**

Catani M, Jones DK, Donato R, Ffytche DH (2003). Occipito-temporal connections in the human brain. Brain 126:2093-2107.

Cox SR, Ferguson KJ, Royle NA, Shenkin SD, MacPherson SE, MacLullich AMJ, Deary IJ & Wardlaw JM (2014) A systematic review of brain frontal lobe parcellation techniques in Magnetic Resonance Imaging. Brain Struct Funct 219:1-22.

Desikan RS, Ségonne F, Fischl B, Quinn BT, Dickerson BC, Blacker D, Buckner RL, Dale AM, Maguire RP, Hyman BT, Albert MS, Killiany RJ (2006) An automated labelling system for subdividing the human cerebral cortex on MRI scans into gyral based regions of interest. NeuroImage 31:968-980.

Forkel SJ, Thiebaut de Schotten M, Kawadler JM, Dell’Acqua F, Danek A, Catani M (2014) The anatomy of fronto-occipital connections from early blunt dissections to comtemporary tractography. Cortex 56:73-84.

Rajkowska G, Goldman-Rakic PS (1995) Cytoarchitectonic definition of prefrontal areas in the normal human cortex: II. Variability in locations of areas 9 and 46 and relationship to the Talairach Coordinate System. Cereb Cortex 5:323-337.

*Table S2*. Associations between brain cortical thickness and surface area per region.

|  |  |  |  |
| --- | --- | --- | --- |
|  |  | ***r*** | ***p*** |
| Frontal Pole | L | **-0.218** | **<0.001** |
|  | R | **-0.164** | **<0.001** |
| DLPFC | L | **-0.241** | **<0.001** |
|  | R | **-0.318** | **<0.001** |
| IFG | L | **-0.121** | **0.003** |
|  | R | **-0.146** | **<0.001** |
| Lateral Orbitofrontal | L | **-0.375** | **<0.001** |
|  | R | **-0.400** | **<0.001** |
| Medial Orbitofrontal | L | **-0.223** | **<0.001** |
|  | R | **-0.335** | **<0.001** |
| Caudal AC | L | 0.087 | 0.037 |
|  | R | -0.008 | 0.856 |
| Rostral AC | L | **-0.234** | **<0.001** |
|  | R | -0.050 | 0.230 |
| Caudal Middle Frontal | L | **-0.098** | **0.019** |
|  | R | -0.030 | 0.466 |
| Precentral | L | **-0.250** | **<0.001** |
|  | R | **-0.227** | **<0.001** |
| Paracentral | L | -0.003 | 0.938 |
|  | R | -0.035 | 0.403 |
| Postcentral | L | **-0.126** | **0.003** |
|  | R | **-0.117** | **0.006** |
| Insula | L | **-0.094** | **0.024** |
|  | R | **-0.114** | **0.006** |
| STG | L | -0.045 | 0.284 |
|  | R | -0.015 | 0.720 |
| Middle Temporal | L | 0.078 | 0.063 |
|  | R | 0.024 | 0.563 |
| Inferior Temporal | L | -0.008 | 0.847 |
|  | R | -0.003 | 0.943 |
| Temporal Pole | L | 0.021 | 0.622 |
|  | R | -0.024 | 0.572 |
| Entorhinal | L | -0.026 | 0.538 |
|  | R | **-0.203** | **<0.001** |
| Fusiform | L | 0.081 | 0.054 |
|  | R | 0.019 | 0.654 |
| Parahippocampal | L | **-0.204** | **<0.001** |
|  | R | **-0.180** | **<0.001** |
| Posterior Cingulate | L | -0.028 | 0.502 |
|  | R | **-0.264** | **<0.001** |
| Isthmus Cingulate | L | **-0.454** | **<0.001** |
|  | R | **-0.378** | **<0.001** |
| Superior Parietal | L | **-0.207** | **<0.001** |
|  | R | **-0.140** | **<0.001** |
| Inferior Parietal | L | **-0.195** | **<0.001** |
|  | R | **-0.198** | **<0.001** |
| Precuneus | L | **-0.104** | **0.012** |
|  | R | **-0.127** | **0.002** |
| Supramarginal | L | **-0.252** | **<0.001** |
|  | R | **-0.174** | **<0.001** |
| Medial Occipital | L | **-0.161** | **<0.001** |
|  | R | -0.084 | 0.044 |
| Lateral Occipital | L | -0.070 | 0.092 |
|  | R | **-0.118** | **0.005** |

Pearson’s *r* and *p* values are reported. Significant FDR-corrected values in bold.

*Table S3*. Associations between cortical characteristics and cognitive ageing differences between age 11 and 73.

|  |  | **Volume** | | | **Surface Area** | | | **Thickness** | | |
| --- | --- | --- | --- | --- | --- | --- | --- | --- | --- | --- |
|  |  | ***r*** | ***p*** | ***q*** | ***r*** | ***p*** | ***q*** | ***r*** | ***p*** | ***q*** |
| Frontal Pole | L | -0.072 | 0.088 | 0.176 | -0.056 | 0.180 | 0.255 | -0.018 | 0.669 | 0.985 |
|  | R | -0.050 | 0.239 | 0.331 | -0.022 | 0.598 | 0.702 | -0.034 | 0.426 | 0.985 |
| DLPFC | L | **0.108** | **0.010** | **0.047** | **0.157** | **0.000** | **0.002** | 0.000 | 0.999 | 0.999 |
|  | R | 0.074 | 0.079 | 0.176 | **0.133** | **0.002** | **0.007** | -0.042 | 0.318 | 0.985 |
| IFG | L | **0.107** | **0.011** | **0.047** | **0.129** | **0.002** | **0.009** | 0.004 | 0.930 | 0.999 |
|  | R | 0.058 | 0.171 | 0.272 | **0.096** | **0.023** | 0.053 | -0.024 | 0.567 | 0.985 |
| Lateral Orbitofrontal | L | 0.070 | 0.094 | 0.176 | **0.113** | **0.007** | **0.022** | -0.023 | 0.583 | 0.985 |
|  | R | 0.079 | 0.061 | 0.158 | **0.146** | **0.000** | **0.003** | -0.055 | 0.189 | 0.985 |
| Medial Orbitofrontal | L | 0.001 | 0.987 | 0.987 | **0.090** | **0.032** | 0.065 | -0.059 | 0.163 | 0.985 |
|  | R | 0.041 | 0.328 | 0.432 | **0.170** | **0.000** | **0.001** | -0.070 | 0.096 | 0.985 |
| Caudal AC | L | 0.002 | 0.963 | 0.982 | 0.028 | 0.498 | 0.598 | -0.048 | 0.257 | 0.985 |
|  | R | 0.004 | 0.919 | 0.973 | 0.058 | 0.168 | 0.246 | **-0.087** | **0.039** | 0.834 |
| Rostral AC | L | 0.051 | 0.223 | 0.317 | **0.136** | **0.001** | **0.006** | -0.022 | 0.607 | 0.985 |
|  | R | 0.032 | 0.442 | 0.555 | **0.098** | **0.020** | **0.048** | -0.062 | 0.143 | 0.985 |
| Caudal Middle Frontal | L | 0.002 | 0.963 | 0.982 | 0.028 | 0.498 | 0.598 | -0.048 | 0.257 | 0.985 |
|  | R | 0.004 | 0.919 | 0.973 | 0.058 | 0.168 | 0.246 | **-0.087** | **0.039** | 0.834 |
| Precentral | L | 0.053 | 0.216 | 0.316 | -0.021 | 0.619 | 0.711 | 0.041 | 0.334 | 0.985 |
|  | R | 0.064 | 0.135 | 0.220 | 0.014 | 0.735 | 0.778 | 0.027 | 0.525 | 0.985 |
| Paracentral | L | **0.082** | **0.050** | 0.135 | **0.089** | **0.035** | 0.069 | 0.002 | 0.955 | 0.999 |
|  | R | 0.065 | 0.121 | 0.204 | **0.103** | **0.014** | **0.038** | -0.032 | 0.448 | 0.985 |
| Postcentral | L | 0.067 | 0.116 | 0.202 | 0.034 | 0.429 | 0.539 | 0.020 | 0.638 | 0.985 |
|  | R | 0.070 | 0.105 | 0.188 | 0.050 | 0.243 | 0.330 | 0.015 | 0.728 | 0.985 |
| Insula | L | 0.071 | 0.091 | 0.176 | **0.088** | **0.036** | 0.069 | -0.014 | 0.732 | 0.985 |
|  | R | **0.083** | **0.049** | 0.135 | 0.077 | 0.068 | 0.110 | 0.007 | 0.866 | 0.995 |
| STG | L | **0.132** | **0.002** | **0.014** | **0.122** | **0.004** | **0.015** | 0.027 | 0.523 | 0.985 |
|  | R | **0.122** | **0.004** | **0.024** | 0.076 | 0.072 | 0.110 | 0.053 | 0.208 | 0.985 |
| Middle Temporal | L | **0.150** | **0.000** | **0.008** | **0.175** | **0.000** | **0.001** | 0.019 | 0.659 | 0.985 |
|  | R | **0.134** | **0.001** | **0.013** | **0.148** | **0.000** | **0.003** | 0.035 | 0.403 | 0.985 |
| Inferior Temporal | L | **0.135** | **0.001** | **0.013** | **0.169** | **0.000** | **0.001** | 0.000 | 0.992 | 0.999 |
|  | R | **0.105** | **0.013** | **0.047** | **0.152** | **0.000** | **0.003** | 0.001 | 0.986 | 0.999 |
| Temporal Pole | L | 0.073 | 0.083 | 0.176 | 0.078 | 0.066 | 0.110 | 0.052 | 0.216 | 0.985 |
|  | R | 0.009 | 0.825 | 0.928 | 0.016 | 0.710 | 0.778 | 0.049 | 0.251 | 0.985 |
| Entorhinal | L | **0.106** | **0.012** | **0.047** | **0.111** | **0.008** | **0.023** | 0.025 | 0.551 | 0.985 |
|  | R | **0.127** | **0.003** | **0.018** | **0.129** | **0.002** | **0.009** | 0.046 | 0.280 | 0.985 |
| Fusiform | L | **0.151** | **0.000** | **0.008** | **0.180** | **0.000** | **0.001** | 0.013 | 0.760 | 0.985 |
|  | R | **0.106** | **0.012** | **0.047** | **0.099** | **0.020** | **0.048** | 0.016 | 0.701 | 0.985 |
| Parahippocampal | L | 0.056 | 0.187 | 0.288 | 0.045 | 0.287 | 0.378 | 0.005 | 0.911 | 0.999 |
|  | R | 0.072 | 0.088 | 0.176 | 0.049 | 0.244 | 0.330 | 0.007 | 0.864 | 0.995 |
| Posterior Cingulate | L | 0.028 | 0.506 | 0.621 | 0.011 | 0.800 | 0.815 | 0.025 | 0.549 | 0.985 |
|  | R | 0.074 | 0.079 | 0.176 | **0.093** | **0.028** | 0.060 | -0.020 | 0.637 | 0.985 |
| Isthmus Cingulate | L | 0.022 | 0.599 | 0.719 | -0.017 | 0.681 | 0.766 | 0.035 | 0.401 | 0.985 |
|  | R | -0.016 | 0.707 | 0.830 | -0.001 | 0.983 | 0.983 | -0.013 | 0.766 | 0.985 |
| Superior Parietal | L | **0.104** | **0.014** | **0.047** | 0.077 | 0.067 | 0.110 | 0.019 | 0.649 | 0.985 |
|  | R | **0.103** | **0.015** | **0.047** | **0.088** | **0.038** | 0.070 | 0.007 | 0.861 | 0.995 |
| Inferior Parietal | L | **0.110** | **0.009** | **0.046** | **0.144** | **0.001** | **0.003** | -0.009 | 0.825 | 0.995 |
|  | R | **0.148** | **0.000** | **0.008** | **0.118** | **0.005** | **0.016** | 0.028 | 0.503 | 0.985 |
| Precuneus | L | **0.140** | **0.001** | **0.011** | **0.138** | **0.001** | **0.005** | 0.013 | 0.759 | 0.985 |
|  | R | **0.085** | **0.043** | 0.129 | 0.076 | 0.070 | 0.110 | 0.016 | 0.701 | 0.985 |
| Supramarginal | L | 0.077 | 0.069 | 0.170 | 0.081 | 0.056 | 0.102 | 0.008 | 0.851 | 0.995 |
|  | R | 0.047 | 0.272 | 0.367 | 0.039 | 0.360 | 0.463 | 0.005 | 0.904 | 0.999 |
| Medial Occipital | L | 0.034 | 0.420 | 0.539 | **0.094** | **0.024** | 0.055 | -0.078 | 0.062 | 0.834 |
|  | R | 0.053 | 0.208 | 0.312 | **0.119** | **0.005** | **0.016** | -0.082 | 0.051 | 0.834 |
| Lateral Occipital | L | -0.011 | 0.802 | 0.922 | 0.013 | 0.765 | 0.794 | -0.047 | 0.260 | 0.985 |
|  | R | 0.008 | 0.851 | 0.938 | 0.014 | 0.735 | 0.778 | -0.020 | 0.634 | 0.985 |

*Note*. DLPFC = dorsolateral prefrontal cortex, IFG = inferior frontal gyrus, AC = anterior cingulate, STG = superior temporal gyrus. Bold values indicate significant associations. Pearson’s *r*, *p* values, and FDR-corrected *q* values are reported.


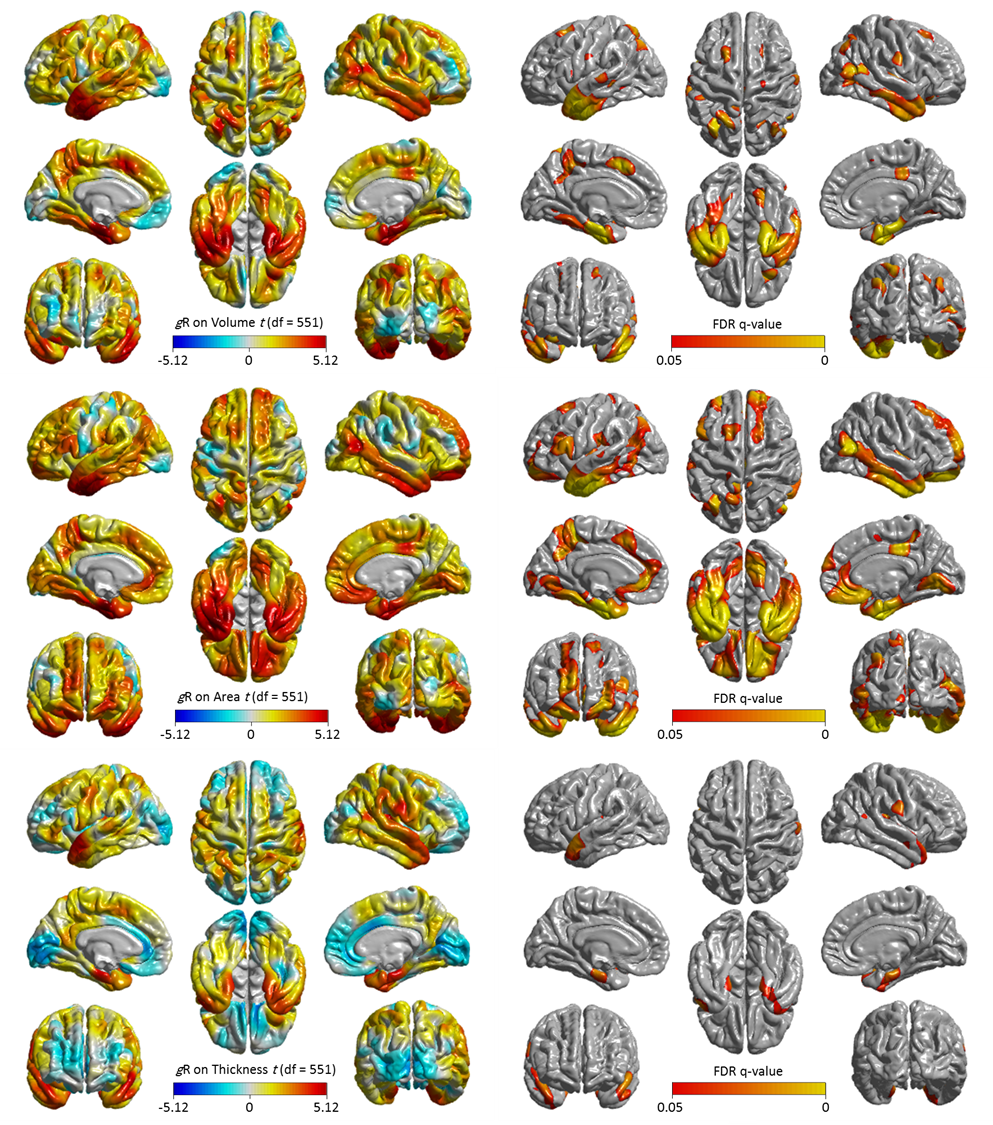
 *Figure S1.* Vertex-wise associations between cortical volume (top row), surface area (middle row) and thickness (bottom row) and cognitive change between age 11 and 73 (corrected for ICV, age and sex). Left hand column reports the t-statistic where hotter colours denote stronger positive associations – e.g. greater surface area associated with less cognitive decline. Right hand column displays FDR-corrected significance.
